# Genome-Wide Identification and Expression Analysis of the STAT Family in Reeve’s Turtle (*Mauremys reevesii*)

**DOI:** 10.1007/s10528-024-10820-7

**Published:** 2024-05-16

**Authors:** Yi Song, Zeshuo Zhou, Shichen Huang, Zhiyuan Li, Xuechi Zhu, Hongming Zhou, Yuxin Jiang, Duminda S. B. Dissanayake, Arthur Georges, Lei Xiong

**Affiliations:** 1 Department of Biochemistry and Molecular Biology, School of Basic Medicine, Wannan Medical College, Wuhu, 241002 China; 2 Department of Pathogenic Biology and Immunology, School of Medicine, Jiaxing University, Jiaxing, 314001 China; 3 School of Clinical Medical, Wannan Medical College, Wuhu, 241002 China; 4 School of Pharmacy, Wannan Medical College, Wuhu, 241002 China; 5 Institute for Applied Ecology, University of Canberra, Canberra, Australia; 6 School of Biological, Earth and Environmental Sciences, Faculty of Science at the University of New South Wales, Sydney, Australia

**Keywords:** Immunity, Signal transduction, Reptiles, Gene evolution

## Abstract

The *Stat* (signal transducer and activator of transcription) gene family plays a vital role in regulating immunity and the processes of cellular proliferation, differentiation, and apoptosis across diverse organisms. Although the functions of *Stat* genes in immunity have been extensively documented in many mammals, limited data are available for reptiles. We used phylogenetic analysis to identify eight putative members of the *Stat* family (*Stat1-1*, *Stat1-2*, *Stat2*, *Stat3*, *Stat4*, *Stat5b*, *Stat6-1*, and *Stat6-2*) within the genome of *M.* *reevesii*, a freshwater turtle found in East Asia. Sequence analysis showed that the *Stat* genes contain four conserved structural domains protein interaction domain, coiled-coil domain, DNA-binding domain, and Src homology domain 2. In addition, *Stat1*, *Stat2*, and *Stat6* contain TAZ2bind, Apolipo_F, and TALPID3 structural domains. The mRNA levels of *Stat* genes were upregulated in spleen tissues at 4, 8, 12, and 16 h after administration of lipopolysaccharide, a potent activator of the immune system. *Stat5b* expression at 12-h LPS post-injection exhibited the most substantial difference from the control. The expression of *Stat5b* in spleen tissue cellular was verified by immunofluorescence. These results suggest that *Stat5b* plays a role in the immune response of *M.* *reevesii* and may prove to be as a positive marker of an immune response in future studies.

**Supplementary Information:**

The online version contains supplementary material available at 10.1007/s10528-024-10820-7.

## Introduction

Signal transducer and activator of transcription (STAT) proteins are intracellular transcription factors involved in regulating the expression of immune-related genes and regulating growth, apoptosis, and differentiation (Villarino et al. 2017). The *Stat* gene family in vertebrates typically has 5–7 members that are generally divided into six major subfamilies: *Stat1, Stat2, Stat3, Stat4, Stat5,* and *Stat6*. *Stat* genes play an important role in immunity and their molecular structures and biological roles have been extensively studied in mammals, such as humans and mice (Nguyen-Jackson et al. 2010). *Stat1* acts as a major regulator and is involved in antiviral immunity. For example, mice lacking *Stat1* are susceptible to mucosal fungal infections (Jung et al. 2020). *Stat3* is involved in the release of mature neutrophils from the bone marrow to the circulatory system. *Stat3* knockout mice do not efficiently mobilize the release of mature granulocytes compared to normal mice (Pencik et al. 2016; Zhao et al. 2016). *Stat5A* and *Stat5B* are susceptible to activation by various cytokines, such as interleukins (Hennighausen and Robinson 2008). In humans, *Stat6* is involved in the development of lymphoma and leukemia (Bruns and Kaplan 2006). In reptiles, there have been studies on individual members of the STAT family play a crucial role during gonadal differentiation and antiviral responses. In the red-eared slider turtle (*Trachemys* *scripta* *elegans*), calcium concentration affects the phosphorylation status of STAT3, thereby regulating the transcriptional activity of other genes, influencing male gonadal differentiation (Weber et al. 2020). Additionally, the identification of interferon-gamma (IFN-γ) and its receptor (IFN-γR) in the green anole lizard (*Anolis* *carolinensis*) not only reveals the significance of the interferon system in the immune response of reptiles but also highlights the central role of the STAT family in regulating these processes, as IFN-γ signaling typically proceeds through the STAT1 pathway (Ghorai and Priyam 2018). Thus, in reptiles such as turtles there is a lack of information on STAT family members as a whole.

In aquaculture, turtles often suffer from infection-based diseases and inflammatory reactions. Current research focuses on pathogenesis, disease diagnostic methods, therapeutic means, and preventive measures (Johnson et al 2005; Di Ianni et al 2015; Ciccarelli et al 2020). Due to the long lifespan and unique physiological characteristics of turtles, the study of their immune response mechanisms is somewhat complex. Lipopolysaccharide (LPS) is a component of the outer membrane of Gram-negative bacteria that elicits a strong immune response in animals. When researchers inject LPS into a model organism, they are not introducing live bacteria or causing an infection in the traditional sense. Instead, they are simulating the effects of an infection by triggering an immune response similar to what might be seen during an actual bacterial infection. This is often done to study the immune system’s response to pathogens or to test the efficacy of drugs and treatments that target inflammatory processes. In recent years, numerous studies have shown that infections that release LPS leads to various diseases in animals (Pinya et al. 2021; Xu et al. 2021; Hashimoto et al. 2020). In LPS-mediated cellular inflammation models, STATs regulate immune cell differentiation through the Janus kinase/signal transducer and activator of transcription (JAK/STAT) signaling pathway (Yao et al. 2007). This signaling pathway regulates immunity in zebrafish, and *Stat* gene expression was detected in the spleen of blunt-mouthed seabream (Zhang et al. 2018). There is considerably less information available about immunity in reptiles (Zimmerman et al. 2010). *Stat* genes have rarely been studied in freshwater turtles and whether they play a potential role in immune responses requires further exploration.

The Chinese three-keeled pond turtle, *Mauremys* *reevesii*, in the family Geoemydidae, is a freshwater turtle distributed across many East Asian country across China, the Korean Peninsula and Japan (Rhodin et al. 2017). *M.* *reevesii* holds significant commercial importance in aquaculture and is categorized as “Endangered” on the IUCN Red List of Endangered Species (Rhodin et al. 2017). Notably, this species possesses distinctive characteristics, including a remarkable capacity for hypoxia tolerance, temperature-dependent sex determination, and the ability to produce fertile interspecific and intergeneric hybrids (Xiong et al. 2020). These exceptional traits make it a rare and invaluable model species for evolutionary genetic research. However, the study of gene function in this species, including immunity-related genes, remains unclear and vastly underexplored, resulting in limited information about turtles.

This study characterizes the *Stat* genes in the freshwater turtle *Mauremys* *reevesii*, identifies their location in the genome, and demonstrates that one of the members of this gene family, *Stat5b*, responds strongly to administration of lipopolysaccharides (LPS), a potent activator of the immune system. Subcellular localization and expression of *Stat5b* were observed using immunofluorescence (IF). This study provides insight to the potential functional roles of *Stat* genes in regulating the immune response of *M.* *reevesii*.

## Materials and Methods

### Sample Collection

Healthy adult turtles (101.02 ± 6.21 g) were sourced from the East China Turtle Breeding Facility (Wuhu, Anhui Province, China) and were maintained in water at a temperature of 28 °C. The LPS injection experiment followed methods described in a previous study (do Amaral et al. 2002). The turtles were randomly divided into two groups: the treatment group and the control group. In the treatment group, turtles were administered LPS (Sigma, St. Louis, MO, USA) at a dose of 2.5 mg per 100 g of body weight, which was dissolved in a 0.75% NaCl solution. Turtles in the control group were administered the same volume of a 0.75% NaCl solution. Spleen tissues were collected from a total of 30 turtles spread across five time periods – 0-h, 4-h, 8-h, 12-h, and 16-h post-injection (3 infected turtles as a group, *n* = 3)—representing control and treatment classes. The whole experiment was repeated three times to yield a total of 90 samples which yielded sufficient statistical power. All samples were immediately snap frozen in liquid nitrogen and subsequently stored at − 80 °C. All experiments complied with the guidelines and regulations of the Institutional Animal Care and Use Committee of Wannan Medical College in Wuhu, China.

### Characterization of Stat family Genes

The *Stat* gene sequences from the Chinese softshell turtle (*Pelodiscus* *sinensis*), Zebrafish (*Danio* *rerio*), and Chinese crocodile lizard (*Shinisaurus* *crocodilurus*) (Table S1) were used as reference for screening *Stat* family candidate genes within the genome assembly of *M.* *reevesii* via BLASTN analysis (E-value threshold < 1e–5). Homologous sequences of the *Stat* genes of *M.* *reevesii* were aligned using DNAMAN (https://www.lynnon.com/download/, v6.0), and structural domains of the STAT proteins were predicted using Pfam (http://pfam-legacy.xfam.org/, v36.0) (E-value threshold < 1e–5) (Liu and Yu 2011). The conserved regions of STAT proteins were predicted by HMMER 3.3.2 software using default parameters and then validated by SMART analysis. MEME (https://meme-suite.org/meme/doc/meme.html, v5.5.4) and InterProScan (https://www.ebi.ac.uk/interpro/about/interproscan/, V 5.65–97.0) with the default parameters were used to identify conserved motifs of STAT genes (Bailey et al. 2009). The gene structure of *M.* *reevesii* *Stat* genes was analyzed using TBtools (https://github.com/CJ-Chen/TBtools/releases, v1.045) (Chen et al. 2020). *Stat* genes promoter regions (2000 bp from the start codon, supplementary Table 1) were used in JASPAR database (https://jaspar.elixir.no/) as described previously (Wu et al. 2021). Genomicus (https://www.genomicus.bio.ens.psl.eu/genomicus-110.01/cgi-bin/search.pl, v110.01) was used to determine the location of *Stat* genes on chromosomes.

STAT protein sequences for 16 species (*Homo* *sapiens*, *Mus* *musculus*, *Bos* *taurus*, *Gallus* *gallus*, *Meleagris* *gallopavo*, *Danio* *rerio*, *Xenopus* *laevis*, *Gasterosteus* *aculeatus*, *Oreochromis* *mossambicu*, *Chrysemys picta*, *Terrapene mexicana*, *Pelodiscus* *sinensis*, *Chelonia* *mydas*, *Thamnophis* *elegans*, *Alligator* *mississippiensis*, and *Anolis* *carolinensis*) were downloaded from NCBI (Table S2) to which the STAT amino acid sequence for *M.* *reevesii* was added. Phylogenetic analysis (MEGA 11.0 software (https://megasoftware.net/, v11.0) (Edgar 2004)) was used to assign the *Stat* genes of *M.* *reevesii* standard vertebrate classes (*Stat1-6*). ExPASy (https://web.expasy.org/protparam/) and SnapeGene (https://www.snapgene.cn/, v7.0) were used to assess the physiological and biochemical properties of STAT protein sequences. Prediction of 3D protein structure was conducted using the Phyre2 protein structure homology model building program (http://www.sbg.bio.ic.ac.uk/phyre2, v2.0) (Wass et al. 2010).

### Transcriptomics

Total RNA extraction from spleen tissues was done using TRIzol reagent (Invitrogen, Waltham, MA, USA). RNA concentration and purity were determined utilizing a NanoDrop 2000 system (Thermo Fisher, Waltham, MA, USA). Reverse transcription to cDNA was performed with the PrimeScript™ RT Kit (Takara, Shiga, Japan) (Ijaz et al 2022). To assess the relative expression of *Stat* mRNA within spleen tissue samples, quantitative real-time polymerase chain reaction (qRT-PCR) was conducted using the StepOnePlus™ Real-Time PCR System (Thermo Fisher) in conjunction with SYBR Green I Master Mix (Krishnaveni et al 2023). Glyceraldehyde 3-phosphate dehydrogenase (GAPDH) served as the internal control. The qRT-PCR amplification conditions consisted of 40 cycles, including initial denaturation at 95 °C for 30 s, followed by denaturation at 95 °C for 5 s, annealing at 60 °C for 20 s, and extension at 72 °C for 20 s.

Relative expression levels of *Stat* genes were computed using the 2^–ΔΔCt^ method (Schmittgen et al. 2008; Huan et al 2022; Li et al 2023). Single-factor analysis of variance (ANOVA, SPSS 24.0) was used to assess the relative expression levels of *Stat* genes. Additionally, data from all experiments, presented as mean ± standard deviation (SD), were analyzed using single-factor ANOVA via GraphPad Prism9. Results are expressed as mean ± standard error (SE). Statistical significance was determined at α = 0.05.

### Immunofluorescence

*Stat5b* expression was examined through immunofluorescence (IF) analysis (Donaldson 2001) for samples taken 12 h following LPS post-infection. Spleen tissues were categorized into three groups: the treatment group (12 h after LPS/NaCl post-infection), the positive control group (NaCl only), and the negative control group (background check, not antibody applied). The tissues were fixed in 4% paraformaldehyde, dehydrated in gradient alcohol. Paraffin-embedded sections were cut using a microtome (5 μm). Sections were subjected to a heat treatment at 65 °C for 6 h, followed by dewaxing and hydration in graded ethanol. They were then rinsed with PBST (phosphate-buffered saline containing 0.3% Triton X-100 detergent; BSW-005, Biotyscience) and subjected to antigen retrieval with sodium citrate before cooling (Abbas and Alkheraije 2023). After three 5 min washes with PBST, the sections were blocked with 5% bovine serum albumin for 30 min. The treatment and positive control sections were then incubated overnight at 4 °C with STAT5B antibody (A12356, 1:100; Abclonal, Wuhan, China), washed again with PBST, and subsequently exposed to a secondary antibody (AlexaFluor 488 donkey anti-rabbit IgG, Invitrogen) at room temperature for 2 h. The negative control sections were treated the same, but without antibody. Nuclei were stained using 4ʹ,6-diamidino-2-phenylindole (286 nmol/L, Sigma) and fluorescence signals were observed using a laser confocal microscope (SP8, Leica, Wetzlar, Germany).

## Results

### Characterization of Stat family Genes

**Fig. 1:**
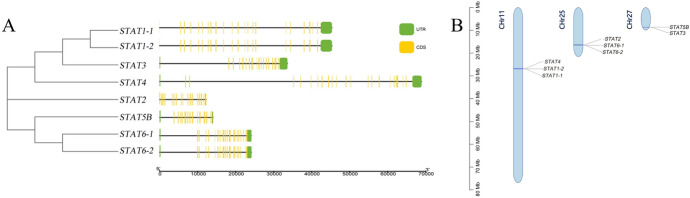
**A** Gene structure analyses of STAT genes in *M. reevesii*. The exons are indicated by yellow rectangles and introns by lines. Yellow rectangles: The sizes of exons number. UTR, untranslated region; CDS, coding sequence (exons). **B** Chromosomal locations of STAT genes in *M. reevesii*

We identified a total of eight *Stat* genes for *M.* *reevesii* (Table 1). Among these genes, *Stat6-1* had the longest amino acid sequence, spanning 861 base pairs, while *Stat1-2* possessed the shortest amino acid sequence at 715 base pairs. In terms of intron count, *Stat1*, *Stat3*, and *Stat4* had the highest number of introns (23 introns), followed by *Stat2* and *Stat6* (21 introns) and then *Stat5b* (18 introns) (Fig. 1A). These genes were distributed across three chromosomes within the *M.* *reevesii* genome—chromosomes 11, 25, and 27. Notably, several pairs of *Stat* genes were found on the same chromosomes, such as *Stat1* and *Stat4* on chromosome 11, *Stat2* and *Stat6* on chromosome 25, and *Stat3* and *Stat5* on chromosome 27. Additionally, some duplicated genes were arranged on the same assembly scaffold, including *Stat1-1/Stat1-2* and *Stat6-1*/*Stat6-2* (Fig. 1B).

**Table 1:** The STAT family genes identified in *M*. *reevesii*

Gene symbol	Accession number	mRNA	Protein	CDS length (bp)	Protein length (aa)	Chromosome location	PI	Instability index	Hydropathicity
*STAT1-1*	120,374,631	XM_039494596.1	XP_039350530.1	2268	756	Chromosome 11	5.54	48.10	− 0.415
*STAT1-2*	120,374,631	XM_039494600.1	XP_039350534.1	2145	715	Chromosome 11	6.19	48.15	− 0.445
*STAT2*	120,391,254	XM_039514731.1	XP_039370665.1	2556	852	Chromosome 25	5.54	47.37	− 0.488
*STAT3*	120,392,259	XM_039516943.1	XP_039372877.1	2316	772	Chromosome 27	5.94	88.25	− 0.354
*STAT4*	120,374,670	XM_039494691.1	XP_039350625.1	2250	750	Chromosome 11	5.97	51.33	− 0.265
*STAT5B*	120,392,261	XM_039516947.1	XP_039372881.1	2349	783	Chromosome 25	5.92	54.66	− 0.472
*STAT6-1*	120,391,151	XM_039514557.1	XP_039370491.1	2583	861	Chromosome 25	5.95	94.33	− 0.242
*STAT6-2*	120,391,151	XM_039514558.1	XP_039370492.1	2490	830	Chromosome 25	6.02	91.32	− 0.238

**Fig. 2:**
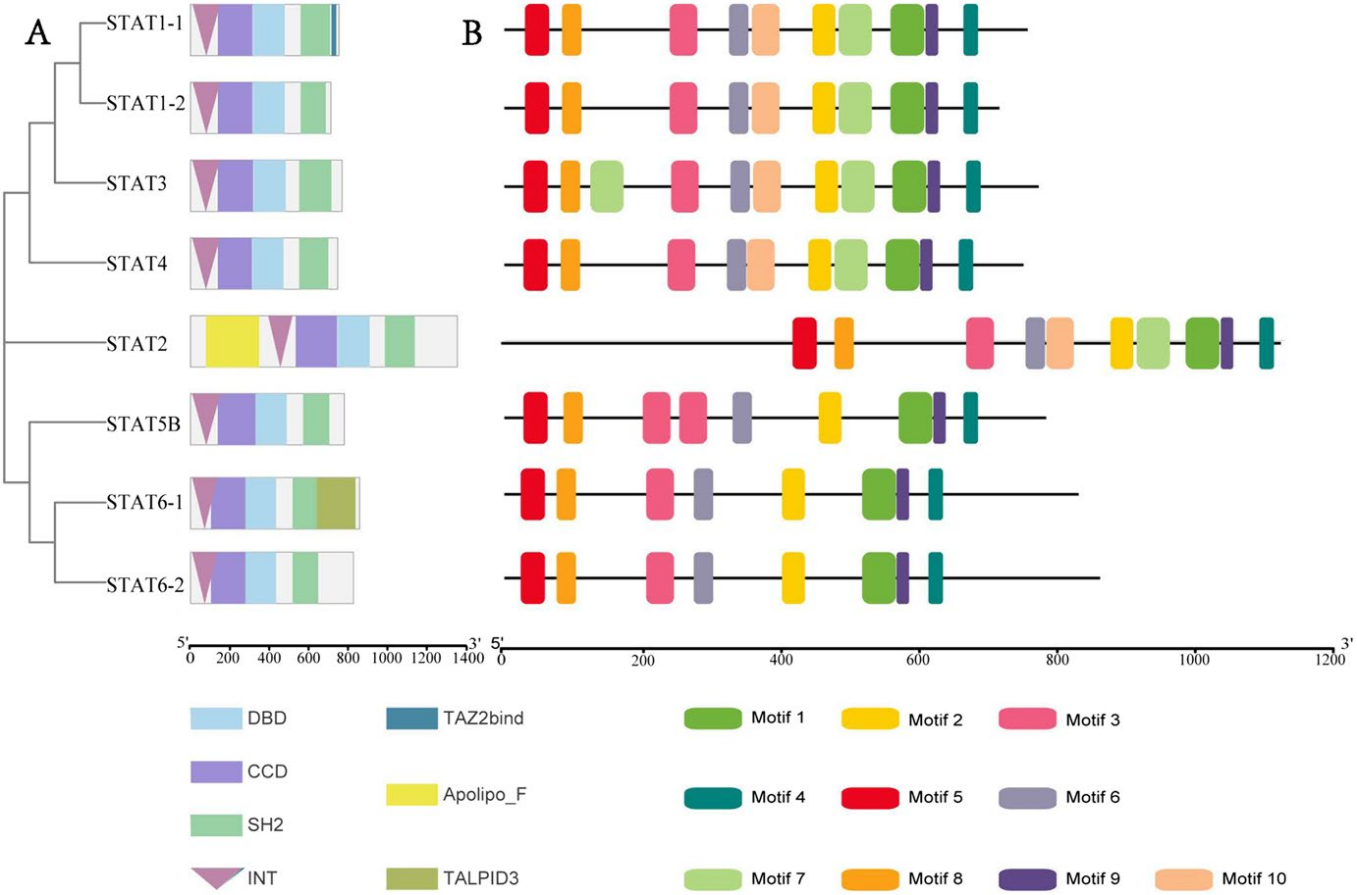
Schematic of the conserved domains in STATs proteins and motifs in STATs genes. All motifs were identified using the Multiple Expectation Maximization for Motif Elicitation database with the complete nucleotide sequences of STATs genes. Gene structure analyses of *Stat* genes in *M. reevesii*. **A** chromosomal locations of *Stat* genes in *M. reevesii* (**B**)

The coding sequences of *Stat* genes ranged in length from 2145 to 2583 base pairs, encoding proteins ranged in size from 715 to 861 amino acids. Additionally, the isoelectric points of these proteins ranged from 5.54 to 6.19. The sequence analysis revealed that all of the *Stat* genes shared four conserved structural domains: the protein interaction domain (INT), coiled-coil domain (CCD), DNA-binding domain (DBD), and Src homology domain 2 (SH2). Sequence analysis showed that STATs contain four conserved structural domains, INT, CCD, DBD, and SH2, in addition to STAT1, STAT2, and STAT6, which contain TAZ2bind, Apolipo_F, and TALPID3 structural domains. Some conserved domains might have special biological functions (Fig. 2A). Furthermore, among these genes, *Stat*1, *Stat*2 and *Stat*6 contained additional structural domains, namely TAZ2bind, Apolipo_F, and TALPID3.

Ten motifs were identified in *Stat1*, *Stat1-2*, *Stat2*, *Stat3*, and *Stat4*. Eight motifs were identified in *Stat5b*, *Stat6-1*, and *Stat6-2*; motifs 7 and 10 were missing. The analysis of motifs showed that motifs 2, 6, and 10 were related to the DBD domain, which combines with the transcriptional initiation region of downstream genes to regulate gene expression. However, motif 10 was not found in the DBD domain of the *Stat5b* and *Stat6*. Motif 3 was related to the CCD domain, which consists of the coiled-coil (alpha) domain of the Stat proteins. This domain is a region that interacts with other proteins. Motifs 1, 4, and 9 were related to the SH2 domain, which is involved in signal transduction. Motifs 5 and 8 corresponds to the INT domain, which is a protein interaction domain. Motifs 3 and 7 appeared twice in *Stat3* and *Stat5b*, respectively (Fig. 2B). The potential binding sites for transcription factors (TFs) in the stat genes promoter were investigated by JASPAR database (supplementary Table 3). Among these TFs, TATA-box, and MYC have been reported as TFs that regulate the stat genes expression (Gergely et al 2024).

**Fig. 3:**
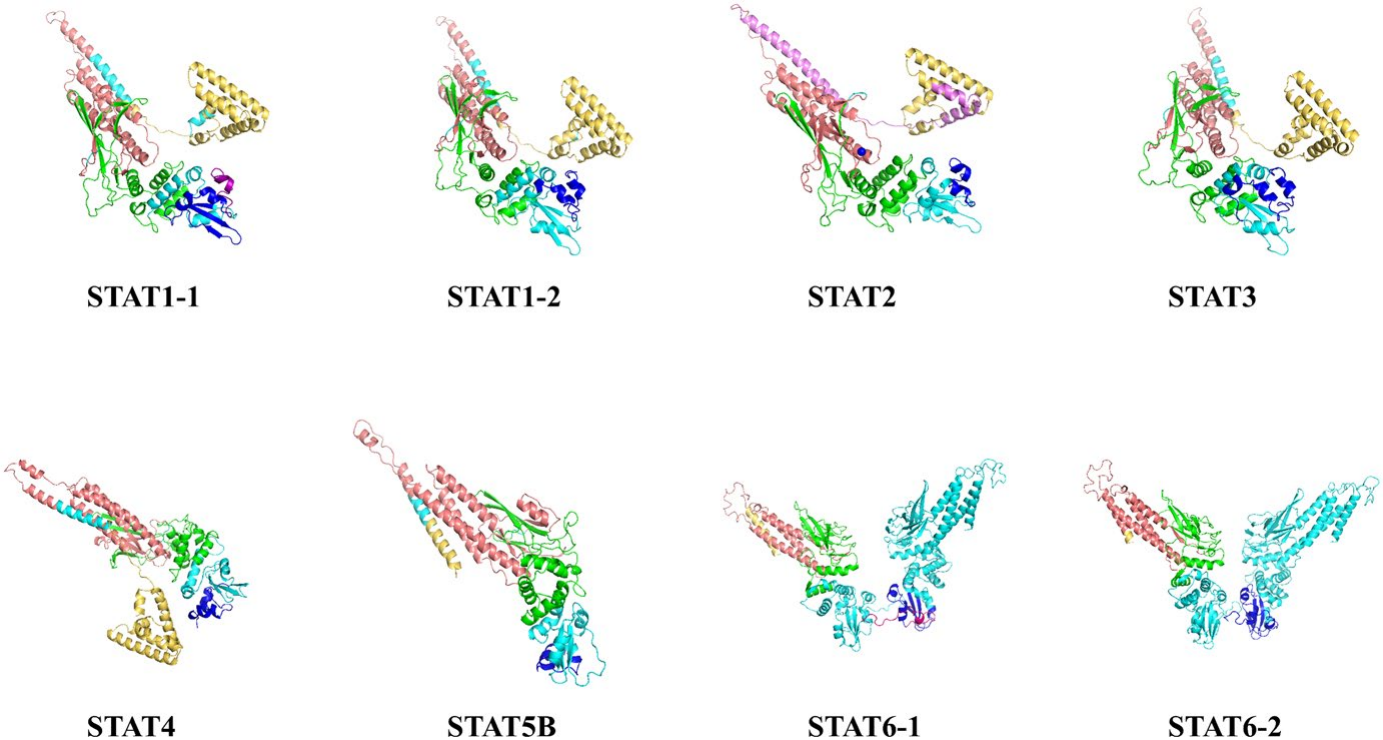
Protein structure of the *Stat* gene from *M. reevesii*. Different colors mark conserved domains

**Fig. 4:**
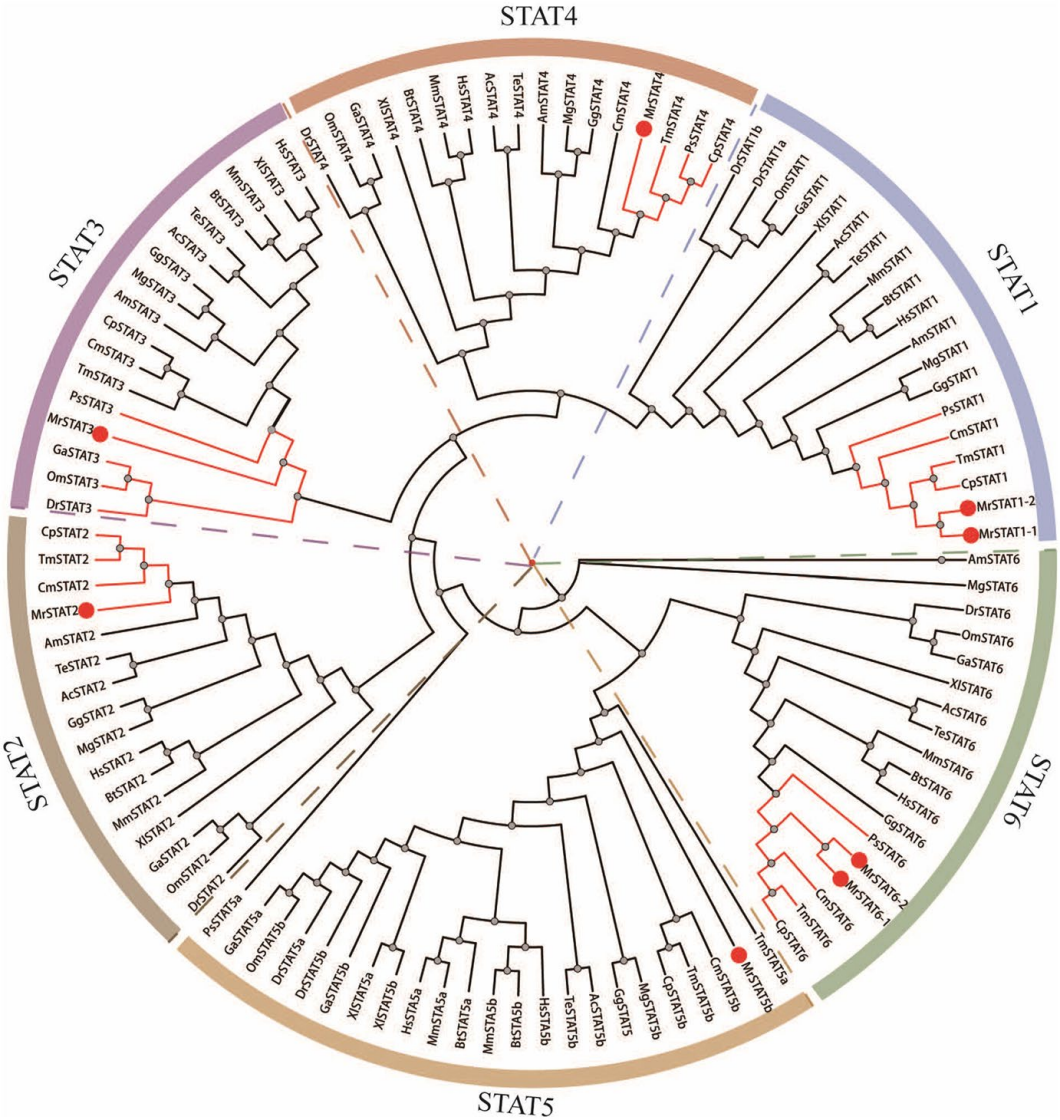
Phylogenetic analysis and classification of STATs homologs from *M. reevesii* and 16 other species. Red circles mark the STATs proteins in *M. reevesii*. Red lines represent the close relationship between STATs and the corresponding homologs

The protein sequences of *Stat* genes were employed for predicting protein structures (Fig. 3). Furthermore, an unrooted phylogenetic tree was constructed using STAT protein sequences from a total of 17 different species (Fig. 4). These homologous sequences were categorized into distinct subfamilies, namely *Stat1*, *Stat2*, *Stat3*, *Stat4*, *Stat5*, and *Stat6*. *M. reevesii* demonstrated its closest genetic affinity with other turtle species, including *C.* *picta*, *T.* *mexicana*, *C.* *mydas*, and *P.* *sinensis*. The subfamilies were organized into two primary cluster groups: Group I encompassed fish *Stat*s, while Group II comprised reptile, mammalian, and avian *Stat*s. The comparative analysis of *Stat* gene locations is presented in Supplementary Table 2.

It is noteworthy that the composition of the Stat family members demonstrated significant interspecies variability. Particularly, the *Stat1* and *Stat6* subfamilies exhibited the highest gene diversity. For instance, species, such as *D.* *rerio*, *T.* *mexicana*, *C.* *mydas*, and among others, featured the coexistence of both *Stat1A* and *Stat1B* paralogs. In contrast, within the context of *M.* *reevesii*, only the presence of *Stat5b* was identified.

### Expression of Stats After LPS Infection

**Fig. 5:**
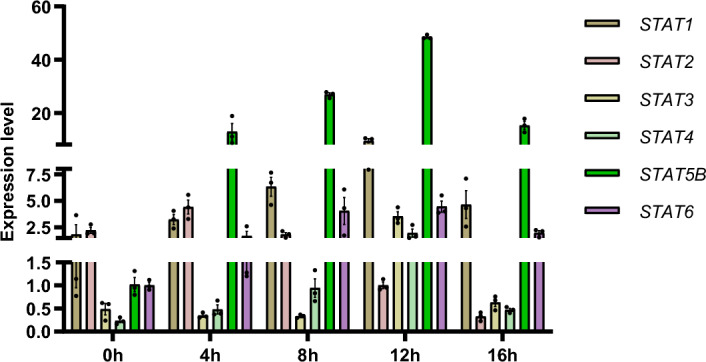
Expression of STAT genes in spleen of post-injection and control turtles at different time points in *M. reevesii*. The whole experiment was repeated three times to yield a total of 90 samples. The experiment was conducted in triplicate and repeated three times. The results were expressed as mean±standard error, *indicates statistical significance at *P*  < *0.05* and **indicates statistical significance at *P*  < *0.01*

All of the *Stat* genes were upregulated compared with the control group (0 h). The expression levels of all genes except *Stat2* and *Stat3* increased rapidly from 0 to 4 h and peaked at 12 h after infection, with a subsequent gradual decrease until 16-h post-infection. Notably, *Stat5b* displayed significantly upregulated expression compared to the other genes after LPS infection, gradually increasing up to 12 h after infection and then decreasing at 16 h. However, *Stat5b* exhibited a significant increase compared to other *Stat* genes (Fig. 5).

### Validation of Expression of Stat5B by Immunofluorescence

**Fig. 6:**
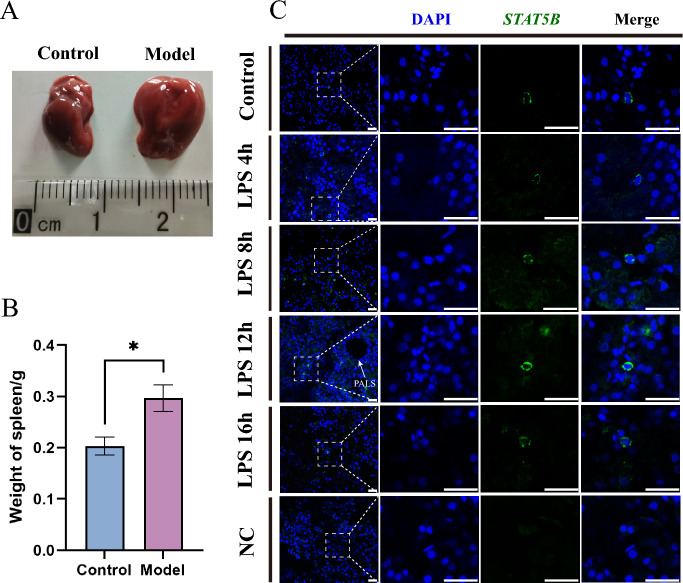
**A** Morphological changes of spleen. **B** Weight statistical analysis of control spleen tissues and LPS post-injected spleen. The experiment was conducted in triplicate and repeated three times. *indicates statistical significance at *P*  < *0.05*. **C** Cellular localization and expression of *STAT5B* in spleen tissue after immune response. The first vertical of line of DAPI was in the cell nucleus. Original magnification × 100. Bar  =  50 μm. PALS, periarterial lymphatic sheath (white arrow). Control means without LPS injection, Model means LPS post-injection 12 h, NC (normal control), treated with antibody diluent solution instead of antibody to determine whether there is non-specific staining in the positive results. Green fluorescence represents the gene signal (*STAT5B*). Original magnification × 400. Bar = 200 μm. Blue fluorescence represents the DAPI signal. The experiment was conducted in triplicate and repeated three times

The spleen of *M.* *reevesii* exhibited a significant increase in both size and weight following LPS infection (Figs. 6A, B). This phenomenon may be attributed to compensatory hyperplasia of the spleen, triggered by the immune response provoked by LPS. In the control group, *Stat5b* was localized within the cytoplasm but was not expressed within the nucleus of spleen cells. However, in the LPS-treated group, *Stat5b* was observed in both the cytoplasm and the nucleus of spleen cells. Conversely, no relevant expression of *Stat5b* was detected in the negative control group. Additionally, the number of *Stat5b-*positive cells in the LPS-treated group demonstrated a marked increase in comparison to the control group. This observation suggests the involvement of *Stat5b-*positive cells in the LPS-induced immune response (Fig. 6C).

## Discussion

The findings from our study shed light on the characterization and expression patterns of *Stat* family genes in the freshwater turtle species *M.* *reevesii*. These genes have pivotal roles in orchestrating diverse and fundamental cellular processes in tetrapods, including immunity, cell proliferation, differentiation, and apoptosis (Levy and Darnell 2002). We identified eight *Stat* genes distributed across three chromosomes. Some genes in the same *Stat* family reside on the same chromosomes suggesting gene duplication or close evolutionary relationships—a phenomenon observed in various species where *Stat* duplications have evolved into novel genes through rapid sequence diversification and neofunctionalization (Wang and Levy 2012). Specifically, *Stat1-1,* *Stat1-2,* and *Stat4* occurred on chromosome 11, *Stat2*, *Stat6-1,* and *Stat6-2* on chromosome 25, and *Stat3* and *Stat*5*b* on chromosome 27. This is unusual and sets *M.* *reevesii* apart from the conventional distribution of *Stat* genes seen in other vertebrates.

Our comparative analysis of *Stat* gene locations in *M.* *reevesii* and other reptilian species, whose chromosome-level assemblies enabled the comparison of gene locations, has unveiled intriguing patterns of genomic organization and evolutionary adaptation. The conservation of specific *Stat* genes on chromosomes across species hints at shared ancestral characteristics and the preservation of crucial signaling pathways. Notably, both *M.* *reevesii* and *C.* *mydas* exhibit the co-location of *Stat1* and *Stat4* on Chromosome 11, suggesting a potential common evolutionary history or functional requirement for these genes in immune responses and cellular regulation in both species. Furthermore, the presence of *Stat3* on Chromosome 27 in both *M.* *reevesii* and *C.* *mydas* raises questions regarding the significance of this genomic arrangement. *Stat3* is renowned for its pivotal roles in signal transduction and immune responses across species and its shared location may imply conserved functions in the immune systems of these reptilian species. This shared gene organization could offer insights into their adaptive responses to environmental challenges. However, the comparative analysis also highlights distinct genomic features that set *M.* *reevesii* apart from other species. Unlike *C.* *mydas*, *A.* *carolinensis*, and *T.* *elegans*, *M.* *reevesii* possesses multiple *Stat6* genes (*Stat6-1* and *Stat6-2*) on Chromosome 25. This unique arrangement suggests that *M.* *reevesii* may have undergone different genomic adaptations, possibly driven by its ecological niche or environmental pressures. These adaptations could be associated with many immune responses and cellular functions to cater to the species’ unique requirements.

One of the most notable findings from our study was the interspecies variation in the number of *Stat* family members. Specifically, the *Stat1* and *Stat6* subfamilies exhibited the highest gene diversity. For instance, species such as *D.* *rerio*, *T.* *Mexicana*, *C.* *mydas*, and others featured both *Stat1a* and *Stat1b* paralogs, suggesting potential functional specialization or redundancy within these genes. In contrast, *M. reevesii* was found to possess only *Stat5b* in this context, implying a unique genetic composition within this species. Comparing *Stat* gene distribution across species reveals both shared patterns and unique arrangements. In birds like *G.* *gallus* and *M.* *gallopavo*, *Stat1* and *Stat4* genes co-locate on Chromosome 7, potentially signifying their significance in avian immune responses. Conversely, *M.* *reevesii* displays a distinct organization with *Stat1-1*, *Stat1-2*, and *Stat4* on Chromosome 11. When examining mammals, humans and cattle share the Chromosome 2 location for *Stat1* and *Stat4*, while *M.* *musculus* differs with Chromosome 1. These differences in genomic organization may be indicative of the species’ distinct evolutionary history or adaptation to specific environmental pressures, leading to such genomic divergence. Continuing our investigation into the functional consequences of these genomic variations will play a pivotal role in unraveling the complex interactions between genomic organization, evolutionary history, and ecological adaptations that underlie the immune responses of these turtle species.

*Stat* genes following LPS infection were significantly upregulated at various time points when compared to the control group. The most pronounced up-regulation occurred at the 12 h followed by a gradual decline until 16-h post-infection, signifying the involvement of *Stat* genes in the immune response to LPS infection. Up-regulation was greatest in *Stat5b* suggesting a central role in the immune response. Our immunofluorescence analysis of the *Stat5b* gene, coincident with a substantial increase in spleen size and weight following LPS infection, suggests compensatory hyperplasia driven by the immune response. *Stat5b*, originally localized in the cytoplasm but absent in the nucleus of spleen cells in the control group, translocated to both cellular compartments in the LPS-treated group. This suggests a role also for *Stat5b* in regulating immune responses at the cellular level.

*Stat5b* is a critical component of the Janus kinase (JAK)-signal transducer and activator of STAT pathway, which is pivotal in transmitting signals from cytokine receptors to the nucleus, thus influencing gene expression (Hashimoto et al 2020). The growth hormone (GH)/insulin-like growth factor (IGF) axis may play a role in fish immune regulation and the Tyrosine-protein kinase (JAK2)/STAT5B pathway activated by GH/IGF signaling. *Stat5b* is predominantly expressed in the liver and responds to growth factor, GH has been shown to stimulate phosphorylation of JAK2 and activation of STAT5B transcriptional activity (Ahmed and Farquharson 2010), and GH-induced expression of hepatic IGF-1 genes is reduced upon loss of *Stat5b* function (Lau-Corona et al. 2017). In this study, We also enriched the JAK/STAT5B pathway from our previous gonadal transcriptomic data of *M. reevesii* (Xiong et al 2019) (Supplementary Fig. 1), although this was not the tissue after being injected with LPS, the expression of related genes on this pathway, such as growth hormone receptor (*GHR*), *SOCS2* (Suppressor of cytokine signaling 2), *JAK2*, and *IGF-1*, showed significant differential expression (Supplementary Fig. 2) between the ovaries and testes, suggesting that JAK/STAT5B pathway is highly sensitive, and whether it actively participates in the immune response after LPS post-injection deserves further investigation.

The realm of freshwater turtle immunity relatively underexplored due to the non-model status of these organisms, which presents a significant lack of genomic information and coordination in the study of their cellular functions. This inherent challenge has hampered our understanding of their immune systems. However, previous studies have unearthed key components such as polymeric immunoglobulin receptors and Toll-like receptors in the innate immunity of the Chinese soft-shelled turtle (Xu et al. 2021; Hashimoto et al. 2020; Yao et al. 2007).

In our current study, we conducted an in-depth analysis that led to the identification and characterization of eight Stat genes within the genome of *M. reevesii*. This comprehensive examination allowed us to gain insights into the structural domains and potential binding sites of these Stat genes, ultimately shedding light on their functional roles. Notably, these structural domains have been conserved across species and are likely to carry specific biological functions. For instance, the TAZ2bind domain, identified in Stat genes, has been demonstrated to function as a transcriptional activator capable of initiating the transcription of specific genes, as documented in previous studies (Kim et al. 2022). Additionally, the TALPID3 domain, another conserved feature of these Stat genes, has been associated with the binding of target genes within the Hedgehog pathway, a critical pathway involved in protein anchoring (Beachy et al. 2010; Ben et al. 2011). These findings provide substantial scientific evidence supporting the functional significance of these conserved domains within the *Stat* genes of *M. reevesii*.

The substantial up-regulation of *Stat5b* in LPS-infected cells strongly implies its active participation in the immune response. Nonetheless, the precise cell type responsible for this response remains to be identified. It might be possible to isolate the different immune cell subpopulations by cell sorting techniques and perform functional assays to determine which cell types exhibit up-regulation of Stat5b after LPS stimulation and show altered immune cell function. Nevertheless, *Stat5b* presents itself as a promising candidate for a potential immune response marker. This research contributes significantly to our expanding comprehension of immune regulation in non-model organisms, such as freshwater turtles, and underscores the importance of further exploration in this domain.

## Conclusion

Our study offers comprehensive insights into the characterization of *Stat* family genes in *M.* *reevesii*, emphasizing their structural diversity, potential functions, and evolutionary relationships. The upregulation of *Stat* genes following LPS infection, with particular emphasis on *Stat5b,* suggests their pivotal role in orchestrating the immune response in this species. These findings contribute to our understanding of the molecular mechanisms underlying immune responses in turtles and provide a foundation for future research in this field.

## Supplementary Information

Below is the link to the electronic supplementary material.

Supplementary file1 (TIF 53812 kb)

Supplementary file2 (TIF 6882 kb)

Supplementary file3 (XLSX 16 kb)

Supplementary file4 (XLSX 18 kb)

Supplementary file5 (XLSX 11 kb)
