# Supplementary figures and images for: Genome-Wide Identification and Expression Analysis of the STAT Family in Reeve’s Turtle (Mauremys reevesii)
Source: Biochem Genet. 2024 May 16;63(3):2330–46. doi: 10.1007/s10528-024-10820-7 (PMC12144074; doi:10.1007/s10528-024-10820-7)

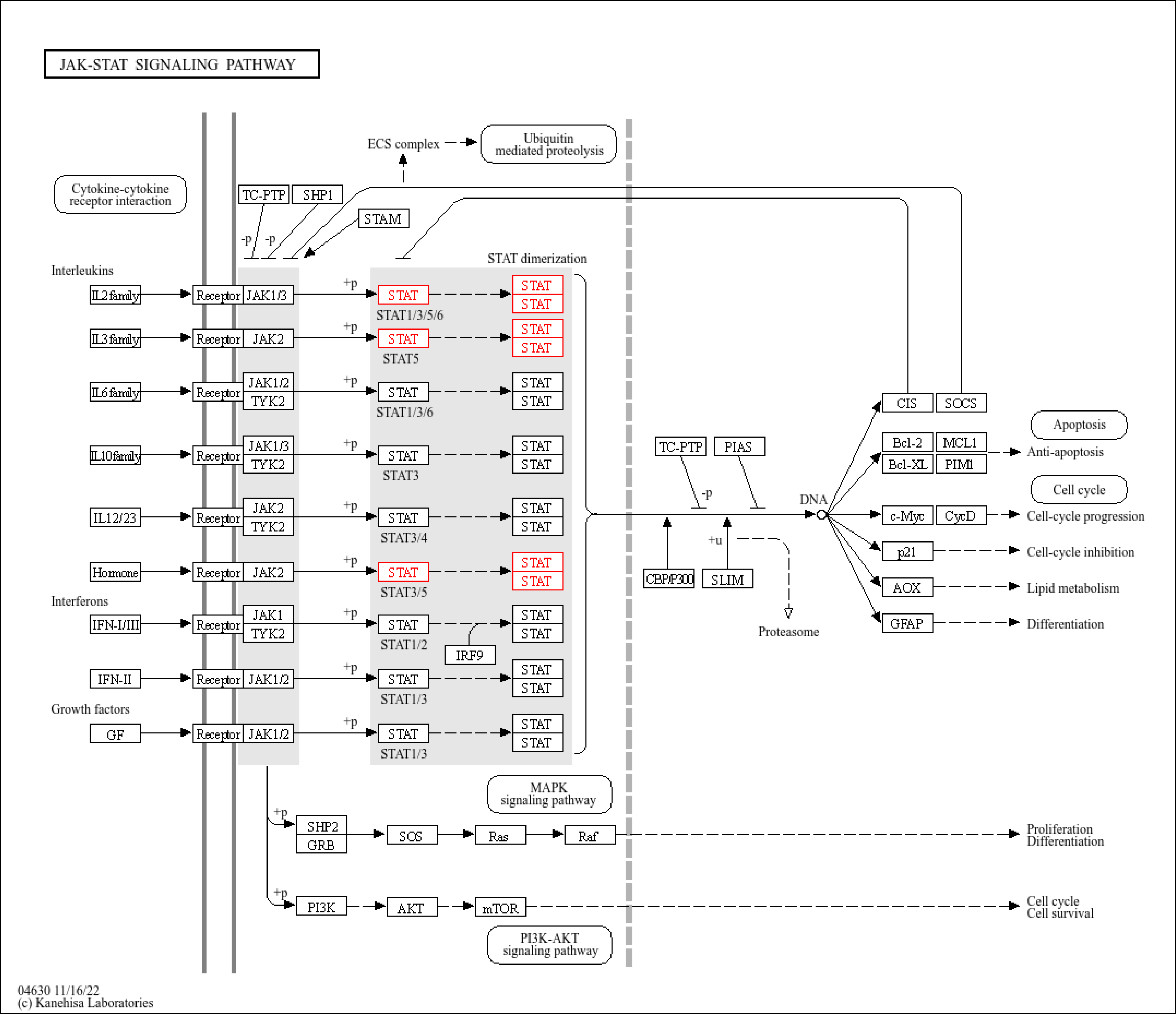

Supplement: Supplementary file 1 — Supplementary file1 (TIF 53812 kb) [file 10528_2024_10820_MOESM1_ESM.tif]

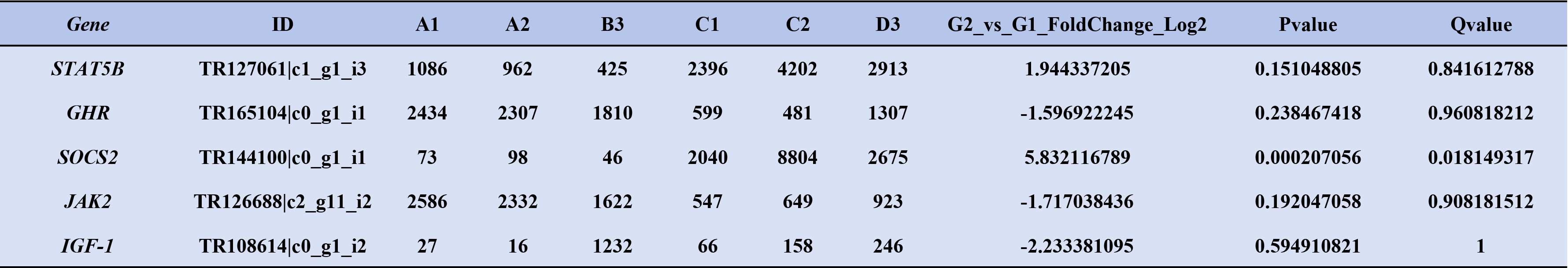

Supplement: Supplementary file 2 — Supplementary file2 (TIF 6882 kb) [file 10528_2024_10820_MOESM2_ESM.tif]
